# X‐Linked Sideroblastic Anaemia Caused by Intronic *ALAS2* Variant Resulting in Highly Variable Expressive Phenotype in Male Siblings, a Case Report

**DOI:** 10.1002/jha2.70060

**Published:** 2025-05-19

**Authors:** James O'Connor, Niall Mannion, Caoimhe McKenna, Kerrie Sweeney, Aaron Niblock

**Affiliations:** ^1^ School of Medicine Ulster University Londonderry UK; ^2^ Northern Ireland Regional Genetics Laboratory Belfast UK; ^3^ Haematology department Antrim Area Hospital Antrim UK

**Keywords:** *ALAS2*, case report, congenital sideroblastic anaemia, haematopoietic stem cell transplantation, variable expressivity, X‐linked sideroblastic anaemia, XLSA

## Abstract

X‐linked sideroblastic anaemia (XLSA) is a rare hereditary disorder caused by mutations in the *ALAS2* gene, essential for haem biosynthesis. We report two male siblings, the first of whom developed severe microcytic hypochromic anaemia requiring regular transfusions, iron chelation and an allogeneic bone marrow transplant, while his brother displayed only mild microcytic hypochromic indices without anaemia. Initial genetic screening did not identify a pathogenic variant. However, duo exome sequencing later revealed an intronic *ALAS2* mutation, initially categorised as of uncertain significance and subsequently reclassified as pathogenic. This case underscores the diagnostic challenges posed by intronic mutations and the highly variable expressivity of XLSA, even among siblings.

**Trial Registration**: The authors have confirmed clinical trial registration is not needed for this submission.

## 1 Introduction

Sideroblastic anaemias are a heterogeneous group of inherited and acquired disorders characterised by the presence of ring sideroblasts in the bone marrow, reflecting defective haem biosynthesis [1]. Among inherited forms, X‐linked sideroblastic anaemia (XLSA) is the most common and results from mutations in the *ALAS2* gene located on Xp11.21. This gene encodes 5‐aminolevulinate synthase, the enzyme catalysing the first step in haem biosynthesis [1]. Mutations in *ALAS2* impair enzyme function, leading to reduced mitochondrial haem synthesis. The phenotypic expression of XLSA is highly variable, ranging from mild microcytosis to severe hypochromic microcytic anaemia with complications such as iron overload, ineffective erythropoiesis and splenomegaly [2]. Diagnosis relies on a combination of clinical findings, laboratory tests (e.g., blood counts, iron studies and peripheral smears), bone marrow biopsy with Prussian blue staining for ring sideroblasts and genetic testing [2].

We report two brothers with the same intronic *ALAS2* mutation. Patient 1 presented with severe anaemia requiring blood product support and eventual haematopoietic stem cell transplantation, while Patient 2 exhibited only mild microcytosis. This case highlights the diagnostic challenges posed by intronic mutations and the variable clinical expressivity of XLSA.

## 2 Case Presentation

### 2.1 Patient 1

Patient 1, the younger sibling, first presented to his general practitioner (GP) at the age of 5 for an unrelated reason and was noted to have significant pallor. A full blood count revealed severe microcytic hypochromic anaemia. Initial management with repeated iron supplementation was ineffective. Over the next 5 years, his anaemia progressively worsened, necessitating regular blood transfusions and, as a result, iron chelation therapy. Other common causes of microcytic anaemias, including thalassaemia, were excluded.

A bone marrow biopsy demonstrated erythroblasts with nuclear‐cytoplasmic asynchrony, and Prussian blue staining revealed ring sideroblasts. These findings were consistent with inherited sideroblastic anaemia, but initial genetic testing failed to identify a causative mutation. Due to the severity of the condition and lack of response to pyridoxine, the patient eventually underwent an allogeneic bone marrow transplant.

**FIGURE 1:**
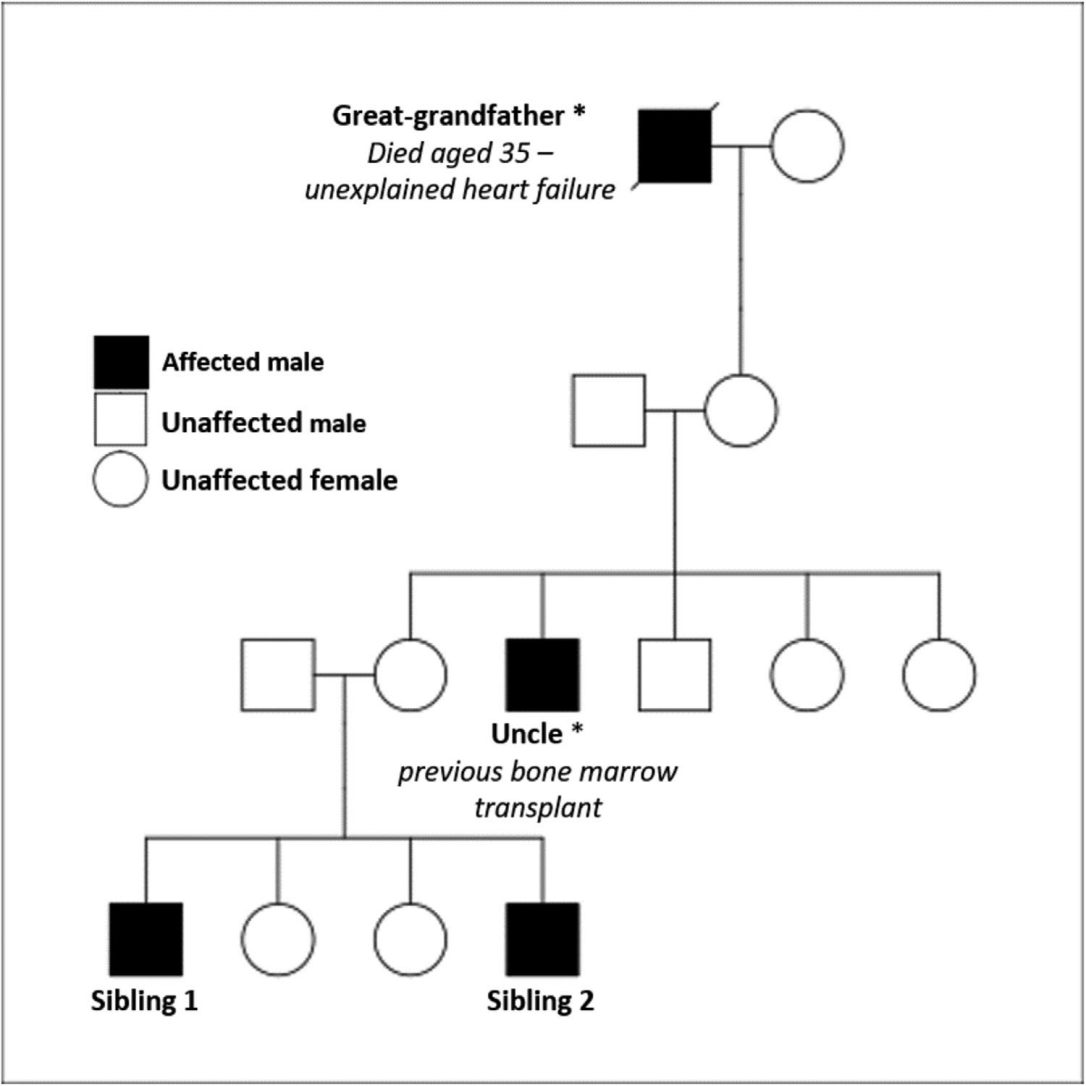
Family pedigree demonstrating presumed X linked mode of inheritance. *the great grandfather and uncle (for technical reasons) were not genetically proven to have the mutation.

Family pedigree analysis (Figure 1) identified an uncle who had received a bone marrow transplant and a great‐grandfather who had died of unexplained heart failure at the age of 35.

### 2.2 Patient 2

Following the diagnosis of Patient 1, his older brother (Patient 2) underwent testing, which showed normal haemoglobin levels but low mean corpuscular volume (MCV) and mean corpuscular haemoglobin (MCH), along with elevated iron and ferritin. Although he exhibited no anaemia, these findings suggested a shared genetic basis. Due to the likely presence of the same underlying mutation, Patient 2 was excluded as a potential bone marrow donor.

Both brothers were found to carry the *HFE C282Y* variant, which may contribute to iron loading but did not fully account for the elevated ferritin levels in such young patients.

### 2.3 Genetic Investigations

Initial screening using a rare anaemia panel did not identify a pathogenic *ALAS2* mutation. However, duo exome sequencing subsequently detected an intronic variant in the *ALAS2* gene in both siblings. This mutation was initially classified as of uncertain significance but was later reclassified as pathogenic following the identification of additional, unrelated cases.

These findings confirmed a diagnosis of XLSA. Despite carrying the same mutation, the brothers exhibited highly variable clinical expressivity, with one being asymptomatic apart from mild microcytosis and the other requiring intensive treatment, including bone marrow transplantation.

## 3 Discussion

Sideroblastic anaemia is characterised by anaemia and the presence of ring sideroblasts in the bone marrow. Congenital sideroblastic anaemia (CSA) is a rare disease caused by mutations in genes involved in the haem biosynthesis. The most prevalent form of CSA is X‐linked sideroblastic anaemia, caused by mutations in the erythroid‐specific δ‐aminolevulinate synthase (*ALAS2*), which is the first enzyme of the haem biosynthesis pathway in erythroid cells [3]. The use of the next‐generation sequencing technology has recognised novel causative genes for CSA [4].

This case emphasises the diagnostic and clinical challenges associated with XLSA. The strikingly variable phenotypes seen in the two brothers underscore the unpredictable expressivity of *ALAS2* mutations.

The phenotypic discordance observed in this case is consistent with prior reports of variability in expressivity of XLSA.

Cazzola et al. reported on two brothers with identical *ALAS2* mutations whereby one brother developed severe anaemia and where the other remained largely asymptomatic [5].

This further supports the posit that even among individuals sharing the same variant of the XLSA mutation, clinical presentation is variable due to potential genetic, epigenetic or external factors.

Initial genetic screening, which focused on exonic regions of the *ALAS2* gene, failed to detect the causative intronic mutation, highlighting a key limitation of current rare anaemia panels. Intronic mutations can impact splicing or gene regulation and should be considered when clinical suspicion of XLSA persists despite initial panel based screening. In this case, duo exome sequencing was crucial for identifying the pathogenic variant [6].

The identification of ring sideroblasts in the bone marrow remains a critical diagnostic hallmark, guiding further genetic investigation. This case also reinforces the importance of considering XLSA in patients with microcytic hypochromic anaemia unresponsive to iron supplementation and pyridoxine, particularly when there is evidence of iron overload at a young age.

Advances in genomic technologies such as exome sequencing are improving our ability to detect noncoding mutations, but these methods are not yet routinely available. This case demonstrates the need for expanded testing in patients with suggestive clinical and laboratory findings.

We also acknowledge that while variability is documented in XLSA, the precise contribution of other genetic or environmental factors remains unknown, and that additional undiscovered factors may contribute to the phenotypic differences observed in this case.

## Author Contributions

A.N. and J.O'C. co‐wrote the manuscript, and with the assistance of N.M. gathered and analysed relevant clinical data. C.McK. reviewed and provided genetic data relevant to this case report. K.S. assisted with development of the family pedigree analysis. This article has been read and approved by all the authors.

## Ethics Statement

The authors have nothing to report.

## Consent

This work was published with consent from the patients.

## Conflicts of Interest

The authors declare no conflicts of interest.

## Data Availability

The data in this study are available from the corresponding author upon reasonable request.
